# Embracing secondary mitral regurgitation with Carillon: past, present, and future

**DOI:** 10.1002/ehf2.13147

**Published:** 2020-12-22

**Authors:** Mitja Lainscak, Michael Böhm

**Affiliations:** ^1^ Division of Cardiology General Hospital Murska Sobota Murska Sobota Slovenia; ^2^ Faculty of Medicine University of Ljubljana Ljubljana Slovenia; ^3^ Klinik für Innere Medizin III, Kardiologie, Angiologie und Internistische Intensivmedizin Universitätsklinikum des Saarlandes Homburg Germany

Mitral regurgitation (MR) is a highly prevalent valvular heart disease.^1^ Several national and international reports have identified MR as a frequent clinical condition in patients with heart failure (HF).^2^, ^3^, ^4^ In many cases, MR is moderate to severe and associated with high mortality and frequent hospitalizations.^4^, ^5^, ^6^ For optimal patient management, it is essential to distinguish between primary and secondary MR, as patient management and interventions differ significantly.^1^

Secondary MR is a disease of the left heart. Left ventricular disease can be due to ischaemic or non‐ischaemic remodelling, whilst atrial disease involving left atrial (LA) enlargement is most often due to elevation of filling pressure leading to atrial fibrillation further promoting LA dilatation.^1^, ^7^ Surgical interventions were developed first to correct secondary MR, but recently, less invasive percutaneous interventions are primarily used.^8^ These include MitraClip, Carillon, Cardioband, PASCAL, Mitralign, and transcatheter mitral valve replacement, with best information available for MitraClip^9^, ^10^, ^11^, ^12^, ^13^ as the most used technique in clinical practice.^14^, ^15^ The findings from clinical trials guiding clinical practice remain inconclusive. Management of secondary MR is a moving target, with both diagnostic and therapeutic challenges. Patients follow a disease trajectory, and it is important when the MR assessment is performed. Next to longitudinal component of worsening of LA and ventricular function, the patient volume status is important, as we generally overestimate MR grade in the congested patient.^1^, ^11^, ^16^, ^17^ Therefore, HF therapies, particularly those that reduce left ventricular end‐diastolic volume or cause reverse remodelling, need to be optimized prior to diagnostic work‐up.^1^, ^18^, ^19^ The crucial role of medical therapy for patient selection and potential effects on trial results was evident in landmark MitraClip trials^9^, ^10^ and should therefore be pursued throughout patient journey. Once patients are considered eligible for mitral valve intervention, the intervention type needs to be selected. Herein, several factors are important including patient, operator, and centre characteristics. Considering the MR prevalence and need for the intervention beyond optimal medical therapy, it is evident that centres will have to perform larger numbers of interventions. In this context, less invasive procedures are needed in day clinic that would be attractive for all partners, including the health care system‐related costs. From the latter perspective, repeated hospitalizations during patient journey are the main driver of financial burden; thus, an early inte

rvention if proven to significantly reduce hospital admissions might be a preferable option (*Figure* *1*).

**Figure 1:**
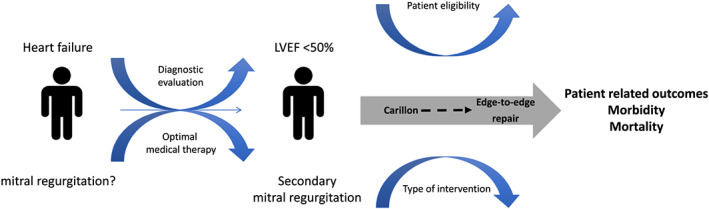
Patient journey of secondary mitral regurgitation in heart failure.

In this issue of the journal, Giallauria and colleagues conducted an individual patient data meta‐analysis of the Carillon mitral contour system.^20^ The device was introduced into clinical practice more than a decade ago, and authors have included 209 patients from three clinical trials^21^, ^22^, ^23^ that compared the Carillon with control. Majority of participants were symptomatic men with left ventricular ejection fraction < 50% and mostly with secondary MR grade 2+ or worse. Analysis focused on echocardiographic parameters to assess severity of MR (mitral regurgitant volume and mitral regurgitant grade) and left ventricular remodelling (left ventricular end‐diastolic and end‐systolic volume). Functional capacity was evaluated with New York Heart Association class. Pooled analysis demonstrated the significant effect of Carillon on all parameters (*P* < 0.005 for all) except on left ventricular end‐systolic volume, for which there was a trend towards reduction (*P* = 0.09). Analysis also included patient outcomes: HF‐related hospitalizations were less common in Carillon group (45% vs. 64%, *P* = 0.04), whereas there was no effect on mortality. Importantly, a sensitivity analysis in MR grade 3+/4+ was performed; it confirmed the observations in the pooled group, with more pronounced reduction in HF‐related hospitalizations (44% vs. 83%, *P* = 0.04).

The Carillon Mitral contour system procedure is relatively quick (usually up to 2 h), safe, and less invasive than other mitral valve procedures.^24^ The device is inserted via the jugular vein into coronary sinus and can be performed by many HF physicians, as most of them have experience with either pacemaker or interventional cardiology procedures. Operator independency is generally reached after three procedures. Some complications were reported, and these include those shared with other interventions (contrast‐induced nephropathy, bleeding, and device dislodgment/fracture). Owing to anatomical relation of coronary sinus with left circumflex artery, extrinsic compression is possible. Coronary sinus venogram and coronary angiogram therefore need to be performed simultaneously at the start of the procedure and prior device release to assess interference with left circumflex artery.

The findings presented by Giallauria and colleagues are, along with original reports,^25^, ^26^ important contribution to the literature and may already be relevant for clinical decisions in HF patients with secondary MR. Owing to less invasive approaches, favourable effects on left ventricular volumes, and possibility of step‐wise upgrade with other interventions, Carillon could be considered early in the patient trajectory for a timely and effective management (*Figure* *1*). Ongoing trials (CARILLON, NCT NCT03142152; AFIRE NCT04529928) are expected to close some of the existing gaps and expand the patient population potentially eligible for secondary MR treatment with Carillon.

## Conflict of interest

M. L. and M. B. received personal fees from Cardiac Dimensions.
